# New California Remedies

**Published:** 1879-02-20

**Authors:** 


### *THE NEW CALIFORNIA REMEDIES.*

Some time ago an article appeared in a Western journal which reflected upon certain of the new remedies from California which are being introduced to the profession by Messrs. Parke, Davis & Co. The article was calculated to destroy confidence in these new agents, and to injure seriously the enterprising drug firm which has expended much labor and invested largely in the work of introducing these medicines to the public. It was stated that the remedies were introduced under fictitious names, &c., and the impression made that they were of little value. It now appears, however, that the editor of the journal referred to, in a recent article, complains that the views of the writer were misinterpreted. The names used are the Spanish names of the medicines, well understood in that country. He concedes that the medicines possess valuable medicinal properties.

